# Improving agroinfiltration-based transient gene expression in *Nicotiana benthamiana*

**DOI:** 10.1186/s13007-018-0343-2

**Published:** 2018-08-25

**Authors:** Karlah Norkunas, Robert Harding, James Dale, Benjamin Dugdale

**Affiliations:** Centre for Tropical Crops and Biocommodities, Queensland University of Technology, Brisbane, QLD 4000 Australia

**Keywords:** Agroinfiltration, pEAQ-HT, Transient, Hyperexpression, *Nicotiana benthamiana*

## Abstract

**Background:**

Agroinfiltration is a simple and effective method of delivering transgenes into plant cells for the rapid production of recombinant proteins and has become the preferred transient expression platform to manufacture biologics in plants. Despite its popularity, few studies have sought to improve the efficiency of agroinfiltration to further increase protein yields. This study aimed to increase agroinfiltration-based transient gene expression in *Nicotiana benthamiana* by improving all levels of transgenesis.

**Results:**

Using the benchmark pEAQ-HT deconstructed virus vector system and the GUS reporter enzyme, physical, chemical, and molecular features were independently assessed for their ability to enhance *Agrobacterium*-mediated transformation and improve protein production capacities. Optimal *Agrobacterium* strain, cell culture density and co-cultivation time for maximal transient GUS (β-glucuronidase) expression were established. The effects of chemical additives in the liquid infiltration media were investigated and acetosyringone (500 μM), the antioxidant lipoic acid (5 μM), and a surfactant Pluronic F-68 (0.002%) were all shown to significantly increase transgene expression. Gene products known to suppress post-transcriptional gene silencing, activate cell cycle progression and confer stress tolerance were also assessed by co-expression. A simple 37 °C heat shock to plants, 1–2 days post infiltration, was shown to dramatically increase GUS reporter levels. By combining the most effective features, a dual vector delivery system was developed that provided approximately 3.5-fold higher levels of absolute GUS protein compared to the pEAQ-HT platform.

**Conclusions:**

In this paper, different strategies were assessed and optimised with the aim of increasing plant-made protein capacities in *Nicotiana benthamiana* using agroinfiltration. Chemical additives, heat shock and the co-expression of genes known to suppress stress and gene silencing or stimulate cell cycle progression were all proven to increase agroinfiltration-based transient gene expression. By combining the most effective of these elements a novel expression platform was developed capable of producing plant-made protein at a significantly higher level than a benchmark hyper-expression system.

**Electronic supplementary material:**

The online version of this article (10.1186/s13007-018-0343-2) contains supplementary material, which is available to authorized users.

## Background

With modern advances in transgenesis and vector design, the use of plant biomass for the cost-effective manufacture of bioproducts continues to improve. Today, transient transformation using *Agrobacterium tumefaciens* is by far the preferred method of protein production as it provides safe, high-level and very rapid transgene expression in comparison to transgenic plants [1–3]. Commonly, the expression cassette containing the gene of interest is carried by recombinant agrobacteria and delivered into the extracellular leaf spaces by physical or vacuum infiltration, a process known as agroinfiltration. In many cases, researchers have relied on a single expression host for this purpose, namely *Nicotiana benthamiana*, because of its amenability to transformation and innate ability to support high levels of heterologous gene expression [4]. While much recent attention has focussed on improving vector design to increase agroinfiltration-based transient expression levels, few studies have sought to address other important aspects of the process as a means of enhancing protein production.

A number of physical factors can influence the efficacy of *Agrobacterium*-mediated transformation including ambient and leaf temperature, light source, pH, osmotic conditions, explant type, bacterial strain and density, and co-cultivation time [5–7]. The design of a suitable artificial environment to promote the interaction of the bacteria and explant is also of considerable importance. The plant-secreted phenolic, acetosyringone, induces virulence gene (*vir*) expression in *Agrobacterium* [8], and the inclusion of this inducer molecule to the co-cultivation media often improves transformation frequencies [9–12]. Acetosyringone is also known to induce the expression of HspL, a small heat-shock protein [13], that is important for VirB protein accumulation and plays a role in promoting *vir*B/D4-mediated DNA transfer [14].

In plant cells, the accumulation of reactive oxygen species (ROS) produced during the oxidative burst response to abiotic stresses or pathogen attack (e.g. *Agrobacterium* infection) can lead to cell damage and necrosis [15–17]. The addition of antioxidant or anti-necrotic compounds such as lipoic acid, ascorbic acid and polyvinylpyrrolidone (PVP) have been shown to delay or inhibit the effects of ROS [18–23]. Similarly, the activation of heat shock proteins *in planta* by exposure to high temperature and the over-expression of gene products known to inhibit apoptosis have also been shown to significantly improve *Agrobacterium*-mediated transformation frequencies, likely by minimising the effects of programmed cell death (PCD) [24]. Plant cell cycle is also of importance as *Agrobacterium* T-DNA delivery reportedly requires a transition through synthesis (S)-phase [25] and the co-expression of a geminivirus-encoded protein with retinoblastoma (RB)-binding activity during *Agrobacterium* infection has been shown to stimulate cell division and increase transformation frequencies [26].

Following T-DNA transfer, transgene expression can be affected by many molecular factors. Transgene mRNA can be rapidly degraded in a targeted, systemic and sequence-specific manner through post-transcriptional gene silencing (PTGS) [27]. This natural plant response to pathogenic or aberrant RNA can drastically reduce transgene expression levels. To overcome this, virus-derived genes such as Tomato bushy stunt virus (TBSV) *p19* and Cucumber mosaic virus (CMV) *2b*, have been co-expressed in order to suppress PTGS [28–30].

Modern plant expression cassettes have been engineered to include virus-derived genetic elements to enhance transcription and translation, amplify gene copy number and suppress PTGS. One such example is the hypertranslatable (HT) vector system [31, 32]. In this vector (pEAQ-HT), transgene expression was controlled by the constitutive Cauliflower mosaic virus (CaMV) 35S promoter and the transgene mRNA was engineered to include translation enhancer sequences derived from Cowpea mosaic virus (CPMV) RNA-2. In addition, the cassette co-expressed the TBSV p19 PTGS suppressor protein. High recombinant protein yields of up to 1.5 g/kg were obtained in *N. benthamiana* using this system [32]. Here, we have assessed the physical, chemical and molecular factors affecting agroinfiltration-based transformation in order to elevate transient gene expression in *N. benthamiana* using the pEAQ-HT vector system and the GUS reporter enzyme. Optimal *Agrobacterium* strain, culture density, and co-cultivation times were determined and the effects of assorted chemical additives in the infiltration media tested. A simple whole plant heat treatment and the co-expression of genes known to suppress stress, PTGS or cell cycle progression were all shown to positively influence recombinant protein accumulation. By combining the most effective of these parameters, a novel protein production platform was developed which provided 3.5-fold higher levels of recombinant GUS protein compared to that of the pEAQ-HT vector system alone.

## Methods

### Reporter gene vector construction

**Fig. 1:**
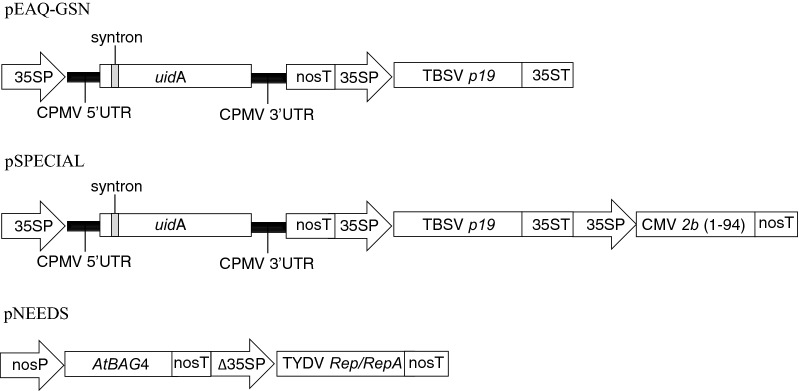
Schematic representation of pEAQ-GSN, pSPECIAL and pNEEDS vectors. pEAQ-GSN was assembled by introducing the *uid*A reporter gene and small synthetic intron (syntron) between the CPMV 5′ and 3′ UTR translation enhancers in pEAQ-HT [31]. pSPECIAL is based on pEAQ-GSN with a downstream expression cassette encoding the truncated CMV 2b (1-94) silencing suppressor protein. pNEEDS is a pBIN-Plus binary vector comprising two expression cassettes encoding the *AtBAG*4 stress tolerance protein under the transcriptional control of the nos promoter and the TYDV Rep/RepA cell cycle control gene products under the transcriptional control of the truncated CaMV (− 90) promoter. 35SP = Cauliflower mosaic virus 35S promoter; CPMV 5′ UTR = Cowpea mosaic virus RNA‐2 5′UTR; *uid*A = gene encoding GUS; syntron = synthetic intron; CPMV 3′ UTR = Cowpea mosaic virus RNA‐2 3′UTR; nosT = nopaline synthase terminator from *Agrobacterium*; TBSV *p19* = Tomato bushy stunt virus *p19* silencing suppressor gene; 35ST = Cauliflower mosaic virus 35S terminator; CMV 2b (1-94) = Cucumber mosaic virus truncated *2b* silencing suppressor gene (amino acids 1-94); nosP = nopaline synthase promoter from *Agrobacterium*; *AtBAG*4=* Arabidopsis BAG*4 gene; ∆35SP = truncated Cauliflower mosaic virus 35S (− 90) promoter; TYDV *Rep*/*RepA *= Tobacco yellow dwarf virus *Rep*/*RepA* gene encoding both Rep and RepA

pEAQ-HT was a generous gift from Sainsbury and Lomonossoff, John Innes Centre, UK [32]. p35S-GSN is a pBIN-Plus vector backbone containing the *uid*A gene (with a small synthetic intron (syntron)) encoding the GUS reporter enzyme under the transcriptional control of the CaMV 35S promoter and nos terminator [33]. The *uid*A gene containing the syntron was excised from p35S-GSN as a *Bam*HI (blunt-ended) and *Sal*I fragment and ligated into *Age*I (blunt-ended) and *Xho*I-digested pEAQ-HT. The resulting construct was called pEAQ-GSN (Fig. 1). All vectors constructed in this study are fully described in Additional file 1.

### Isolation and cloning of genes encoding a stress tolerance protein and suppressors of PTGS

Genomic DNA was isolated from *Arabidopsis thaliana* (cv. Landsberg) using the CTAB method [34] The Bcl-2 associated athanogene 4 (*AtBAG*4) (GenBank Accession NM_115037.7) was amplified by PCR using GoTaq^®^ Green Master Mix, 0.1–1 µg gDNA and 10 µM of the following primer pair At_BAG4-F and At_BAG4-R. PCRs were cycled using the following conditions: 5 min at 94 °C followed by 30 cycles of 94 °C for 20 s, 55 °C for 20 s, and 72 °C for 1 min, with a final extension step of 72 °C for 5 min. All primer sequences used in this study are supplied as Additional file 2.

All PTGS suppressor genes were amplified by PCR from cloned viral components. The TBSV *p19* (M21958.1), CMV *2b* (AB506799.1), Papaya ringspot virus (PRSV) *HC*-*Pro* (JQ394692.1) and Tomato leaf curl virus (TLCV) *TrAP* (NC_003896.1) genes were amplified using the primer pairs TBSVp19-F and TBSVp19-R, CMV2b-F and CMV2b-R, PRSVHCPro-F and PRSVHCPro-R and TLCVTrAP-F and TLCVTrAP-R, respectively. A truncated CMV 2b gene encoding the first 94 amino acids of the CMV 2b protein was initially generated as a PCR anomaly and proven to be as active as the full length 2b gene product. It was re-amplified using primers CMV2b-F and CMV2b-R (1-94).

PCRs were essentially as described above and used 100 ng of plasmid DNA as template. All genes were cloned into pGEM^®^-T Easy (Promega) and confirmed by sequencing using the Big Dye™ Terminator system. All genes were excised from pGEM^®^-T Easy as *AsiS*I and *Sac*I fragments and ligated between the CaMV 35S promoter and nos terminator in similarly digested pBIN-Plus binary vector. These resulting vectors were called p35S-AtBAG4, p35S-TSBV.p19, p35S-CMV.2b, p35S-PRSV.HC-Pro, p35S-TLCV.TrAP, and p35S-CMV.2b (1-94), respectively.

### Isolation and cloning of genes encoding virus-derived cell cycle control proteins

The Tobacco yellow dwarf virus (TYDV) *Rep/RepA* and *RepA* genes were PCR amplified using GoTaq^®^ Green Master Mix, 100 ng of plasmid pDH51 [33] template and primers TYDVRep-Ex1-F and TYDVRepA-R and TYDVRepA-F and TYDVRepA-R, respectively. PCR conditions were as described above. The gene was cloned into pGEM^®^-T Easy (Promega) and confirmed by sequencing using the Big Dye™ Terminator system. The TYDV RepA gene was excised from pGEM^®^-T-Easy using *Eco*RI and *Xba*I and ligated between the CaMV 35S promoter and nos terminator in similarly digested p35S-nos. The resulting vectors were called p35S-TYDV.Rep/RepA and p35S-TYDV.RepA, respectively.

The Banana bunchy top virus (BBTV) *Clink* gene (L41578.1) and upstream CaMV 35S promoter were PCR amplified from p35S‐BBTV.ORF5 using primers 35S‐F and BBTVClink‐R under standard cycling conditions. The PCR product was ligated into pGEM^®^‐T Easy, cloned and sequenced. The resulting vector was called pGEM.35S‐Clink. The CaMV 35S promoter and *Clink* gene were excised from pGEM.35S‐Clink as an *Asc*I and *Xba*I fragment and ligated into similarly digested pBIN‐35S‐nos. The resulting vector was called p35S‐BBTV.Clink.

The Maize streak virus (MSV) *RepA* gene (AY138520) was modified for human codon bias and chemically synthesized by GeneArt^®^ (Life Technologies). Three fragments including (1) a CaMV 35S promoter prepared as an *Asc*I and *Nco*I fragment, (2) the MSV *RepA* gene prepared as an *Nco*I and *Sac*I fragment, and (3) pBIN‐35Snos prepared as an *Asc*I and *Sac*I fragment were assembled in a three-way ligation to generate the vector p35S‐MSV.RepA.

The TLCV *REn* gene (NC_003896.1) and upstream CaMV 35S promoter were PCR amplified from p35SAUSREN [35] using primers 35S‐F and TLCVREn‐R and standard cycling conditions. The PCR product was ligated into pGEM^®^-T Easy, cloned and sequenced. The resulting vector was called pGEM.35S‐REn. The CaMV 35S promoter and *REn* gene from pGEM.35S‐REn were excised as an *Asc*I and *Xba*I fragment and ligated into similarly digested pBIN‐35S‐nos. The resulting vector was called p35S‐TLCV.REn.

Vectors capable of expressing the virus-derived cell cycle genes at low levels were constructed by truncating the CaMV 35S promoter at the –90 position using the unique *Eco*RV restriction site. Vectors p35S-TYDV.Rep/RepA, p35S-TYDV.RepA, p35S-BBTV.Clink, p35S-MSV.RepA and pTLCV-35S.REn were all digested with *Eco*RV and *Pac*I to excise the truncated 35S promoter (Δ35S), the gene of interest and the nos terminator. These cassettes were then ligated into pBIN-Plus digested with *Sma*I and *Pac*I. These constructs were called pΔ35S-TYDV.Rep/RepA, pΔ35S-TYDV.RepA, pΔ35S-BBTV.Clink, pΔ35S-MSV.RepA, and pΔ35S-TLCV.REn, respectively.

Mutation of the LxCxE motif to LxCxK in the TYDV RepA coding region was done using overlapping PCR and the primer pairs: Δ35S-F and TYDV^LxCxK^mut-R; TYDV^LxCxK^mut-F and TYDVRepA-R2. PCR conditions were as described above, and the resulting product ligated into pGEM^®^-T Easy, cloned and sequenced. The mutant gene was excised from pGEM^®^-T Easy as an *Asc*I/*Sac*I fragment and cloned into a similarly digested p35S-GSN backbone. The resulting vector was called pΔ35S-TYDV.RepA^LxCxK^.

### Construction of pSPECIAL and pNEEDS vectors

The 35S-CMV.2b (1-94)-nos gene cassette, encoding the C terminal CMV 2b truncation, was amplified from p35S-CMV.2b (1-94) using primers 35S_FseI-F and nosT_FseI-R and the PCR conditions described above. The resulting product was ligated into pGEM^®^-T Easy, cloned and sequenced. The expression cassette was excised from pGEM^®^-T Easy using restriction enzyme *Fse*I and ligated into *Fse*I-digested and dephosphorylated pEAQ-GSN. The resulting vector was called pSPECIAL (Fig. 1).

The CaMV 35S (-90) promoter, *Rep/RepA* genes and nos terminator cassette was excised from pΔ35S-TYDV.Rep/RepA as an *Eco*RI fragment and ligated into *Eco*RI-digested and dephosphorylated p35S-At.BAG4. To replace the CaMV 35S promoter driving expression of the *AtBAG*4 gene, a nos promoter sequence was PCR amplified with primers nosP_NheI-F and nosP_AsiSI-R from pBIN-Plus plasmid template using the PCR conditions described above. The resulting PCR product was ligated into pGEM^®^-T Easy, cloned and sequenced. The nos promoter was excised from pGEM^®^-T Easy by restriction digestion with *Nhe*I and *AsiS*I and replaced the CaMV 35S promoter upstream of the *AtBAG*4 gene to generate the vector pNEEDS (Fig. 1).

### Agroinfiltration of *N. benthamiana*

Plasmids were mobilized into *A. tumefaciens* strains AGL1, C58C1 and LBA4404 via electroporation [36]. Recombinant agrobacteria were prepared for infiltration using a modified protocol of Sainsbury and Lomonossoff [32]. In short, a single colony of recombinant bacteria was inoculated into liquid LB media (10 g/L tryptone, 5 g/L yeast extract; 10 g/L NaCl, pH 7) or Yeast Mannitol media (0.4 g/L yeast extract, 55 mM mannitol, 2.8 mM K_2_HPO_4_, 800 μM MgSO_4_, 0.1 g/L NaCl, pH 7) containing kanamycin (100 mg/L) and rifampicin (50 mg/L). Cultures were incubated overnight at 28 °C with shaking. Bacteria were pelleted by centrifugation (14,000*g* for 5 min) and resuspended to an OD_600_ = 1.0 in MMA (10 mM MES pH 5.6, 10 mM MgCl_2_, 200 μM acetosyringone) unless otherwise specified. Cultures were then incubated for 2–4 h at room temperature with gentle rocking. Bacteria were delivered into the underside of leaves of 1–2-month-old plantlets using a blunt tipped plastic syringe and applying gentle pressure. For co-transformations, recombinant bacteria containing different plasmids were mixed at a 1:1 ratio immediately prior to infiltration. The top three leaves of three independent plantlets (approximately 6–8 weeks old) were infiltrated with each vector or vector combination. This process was repeated on three separate occasions. Plants were germinated from seed, propagated in growth cabinets at 25 °C with a photoperiod of 16 h and fertilised with Aquasol™ (Yates, a division of DuluxGroup (Australia) Pty. Ltd.) (1 g L^−1^) 2 weeks prior to infiltration.

### Chemical additives and heat shock treatment

Chemicals including lipoic acid (0–100 µM; Merck), ascorbic acid (0–100 mM; Merck), PVP (0–1 g/L; Merck) and Pluronic F-68 (0–0.2%; Thermo Fisher Scientific) were filter sterilized and added to the MMA/bacteria mix immediately prior to infiltration. For acetosyringone, MMA was prepared containing a final concentration ranging from 0 to 600 µM. Chemical additives that were empirically determined to improve the GUS expression levels were combined to form the optimised media MMA-LP (MMA containing 500 μM acetosyringone, 5 μM lipoic acid, and 0.002% Pluronic F-68). Whole plants were heat shocked by placing them in a 37 °C incubator for 30 min, 0–3 days post agroinfiltration.

### Protein extraction and GUS fluorometric assays

*N. benthamiana* leaf samples were collected between 0 and 8 days post agroinfiltration and snap frozen in liquid nitrogen. Total soluble protein (TSP) was extracted by homogenizing the samples in three volumes (w/v) of GUS extraction buffer [38]. The crude lysate was clarified by centrifugation (14,000*g* for 15 min) and protein content estimated using the method of Bradford [37]. GUS enzyme activities were quantified by fluorometric analysis [38] and repeated in triplicate over an enzymatic time course (0, 10 and 20 min). TSP (5 µL) was added to 25 µL of MUG substrate in a microtitre plate and incubated at 37 °C. Reactions were stopped by the addition of 270 µL stop buffer and fluorescence measured using a Perkin Elmer LS50B fluorescence spectrometer (excitation 365 nm, emission 455 nm). Enzyme activities were expressed as μmol 4-MU/mg protein/min.

### GUS ELISA and PAGE analysis

GUS ELISA was performed essentially as described by Dugdale et al. [33]. For PAGE analysis, TSP (20 μg) was electrophoresed through a NuPAGE^®^ Novex^®^ 4–12% Bis–Tris Protein Gel (Life Technologies) at a constant voltage (200 V) for 55 min in NuPAGE^®^ MOPS SDS Running Buffer with NuPAGE^®^ Antioxidant (Life Technologies) according to manufacturer’s specifications. As a control, 0.3 μg of purified GUS protein (GUS Type VII-A; Sigma-Aldrich G7646) was loaded. Protein sizes were estimated using the Novex^®^ Sharp Pre-stained Protein Standard (Life Technologies). Following electrophoresis, the acrylamide gel was stained in Coomassie Brilliant Blue dye overnight (approximately 16 h) and destained in 15% ethanol/10% acetic acid.

### Statistical analysis

Fluorometric GUS measurements from three leaves on three biological replicates over three separate occasions were pooled and the mean calculated. Graphs and basic statistical analysis were generated in Excel; data were expressed as ± the standard error of the mean (SEM). Fluorometric GUS data measurements (in μmol 4-MU/mg protein/min) were converted into a ratio based on the control treatment allowing for comparisons across the different variables tested. Significant differences from the respective controls were calculated using an unpaired *T* test. *p* < 0.05 was considered significant [39].

## Results

### *Agrobacterium* strain and cell density

**Fig. 2:**
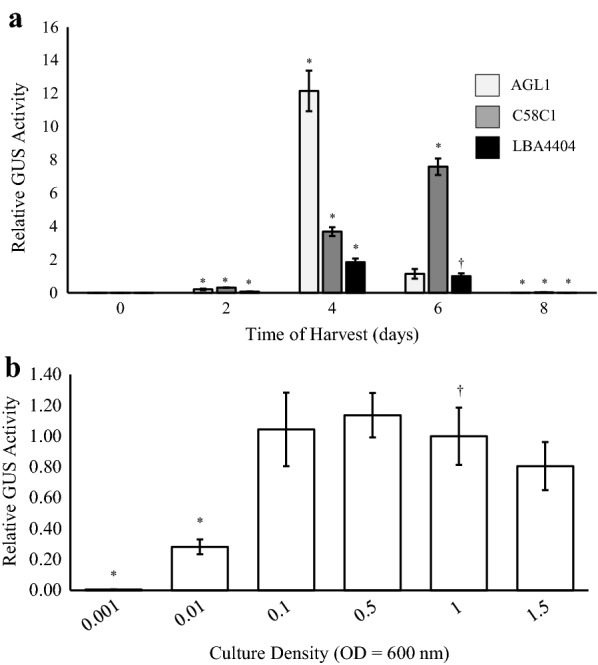
Effects of agrobacteria strain and cell culture density on transient GUS expression via agroinfiltration. **a** Agrobacteria strains AGL1, C58C1 and LBA4404 harbouring pEAQ‐GSN were infiltrated into *N. benthamiana* leaves. Leaves were sampled at 0, 2, 4, 6 and 8 days post infiltration (dpi). **b** *Agrobacterium* strain AGL1 harbouring pEAQ‐GSN was infiltrated into *N. benthamiana* leaves at increasing concentrations OD_600_ = 0.001, 0.01, 0.1, 0.5, 1.0, and 1.5. TSP was extracted for GUS fluorometric enzyme assays. Columns represent relative levels of mean GUS enzyme activities and bars represent ± SE. (†) indicates the reference treatment and (*) indicates data significantly different to the reference (*p* < 0.05)

Three strains of recombinant *A. tumefaciens* (AGL1, C58C1 and LBA4404) harbouring pEAQ-GSN at a concentration of OD_600_ of 1 were infiltrated into the top three leaves of three *N. benthamiana* plantlets and leaves were sampled 0, 2, 4, 6 and 8 days post infiltration (dpi). GUS activity was measured fluorometrically and data from three separate experiments pooled, statistically analysed and graphed (Fig. 2a). Strain LBA4404 at an OD_600_ = 1.0 (6 dpi) was used as the GUS activity reference as these conditions were most similar to those used by Sainsbury et al. [31]. On day 0, negligible levels of GUS expression were observed from all *Agrobacterium* strains suggesting no bacteria-derived or endogenous plant-derived GUS activity. For both strains AGL1 and LBA4404, GUS expression was highest 4 dpi and then decreased to 8 dpi where expression levels were undetectable. For strain C58C1, highest GUS expression was observed at 6 dpi. Highest GUS levels were achieved using strain AGL1 at 4 dpi. This expression level was approximately sixfold higher than that afforded by strain LBA4404 (4 dpi) and about 1.5-fold higher than strain C58C1 (6 dpi).

To examine the effect of agrobacteria concentration on transgene expression, strain AGL1 harbouring pEAQ-GSN was grown to OD_600_ ranging from 0.001 to 1.5 and independently infiltrated into *N. benthamiana*. Leaves were sampled at 4 dpi and GUS activity data from three separate experiments pooled and statistically analysed (Fig. 2b). In comparison to OD_600_ = 1.0, GUS levels were significantly lower using bacterial densities of OD_600_ = 0.001 and 0.01. No significant difference in GUS activity was observed at OD_600_ = 0.1, 0.5 and 1.5. Based on this finding, all subsequent infiltrations used strain AGL1 at a bacterial density of OD_600_ = 1.0. For all subsequent experiments, Day 0 leaf samples were taken immediately after infiltration and GUS activity quantified. Negligible GUS levels were detected in all Day 0 samples tested suggesting no endogenous/background GUS activity (results not shown).

### Chemical additives

**Fig. 3:**
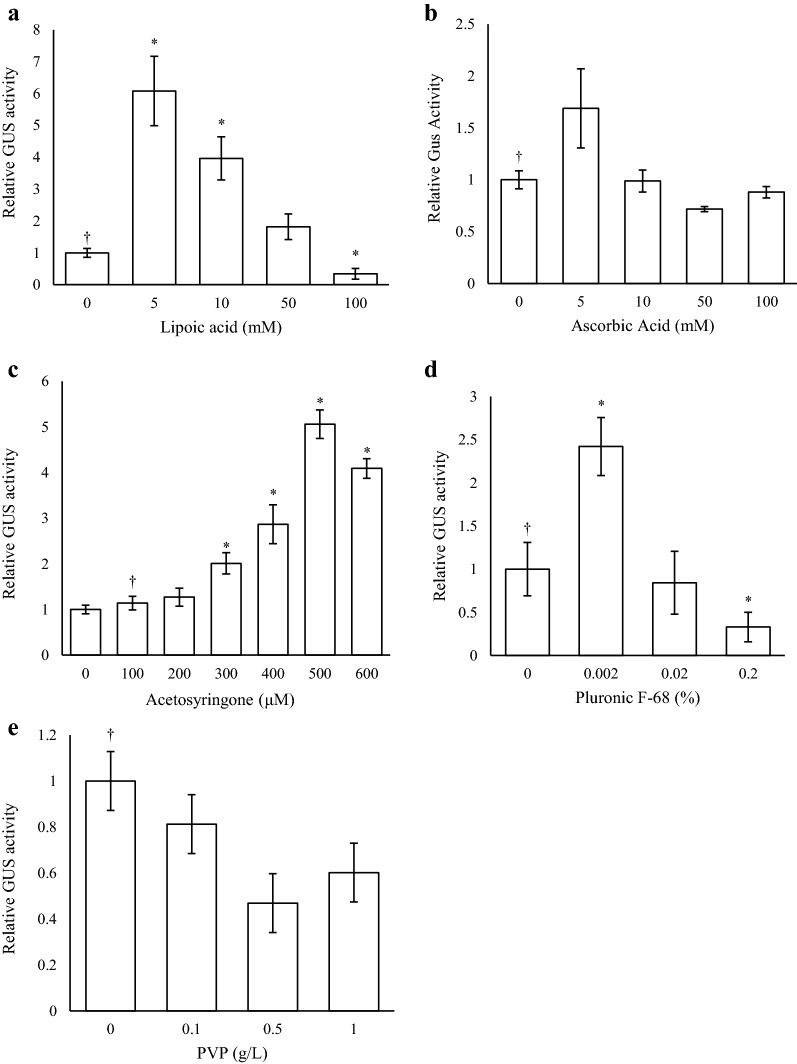
Effects of chemical additives on transient GUS expression. Agrobacteria strain AGL1 harbouring pEAQ‐GSN were infiltrated in MMA media containing different concentrations of chemical additives, **a** Lipoic acid, **b** ascorbic acid, **c** acetosyringone, **d** Pluronic F‐68, and **e** PVP, into *N. benthamiana* leaves. Leaves were sampled 4 dpi and TSP extracted for GUS fluorometric enzyme assays. Columns represent relative levels of mean GUS enzyme activities and bars represent ± SE. (†) indicates the reference treatment and (*) indicates data significantly different to the reference (*p* < 0.05)

The effects of five chemical additives were tested by including these compounds at different concentrations in the MMA co-cultivation media. The addition of lipoic acid at low concentrations of 5 and 10 µM significantly increased GUS levels about sixfold and fourfold respectively, while concentrations above this had no enhancing effect (Fig. 3a). The addition of low levels of ascorbic acid (5 mM) appeared to have a positive effect on GUS activity, although this increase was not statistically significant, and increasing amounts of the anti-oxidant were of no benefit (Fig. 3b). Increasing acetosyringone concentrations correlated with increased GUS levels with greatest activity obtained at a final concentration at 500 μM (Fig. 3c). At this concentration, GUS levels were approximately fivefold higher than those obtained using MMA media alone. For Pluronic F-68, low concentrations (0.002%) increased GUS activity about twofold, while concentrations above this were ineffective (Fig. 3d). Addition of PVP had no stimulatory effects (Fig. 3e).

### Heat shock to whole plants

**Fig. 4:**
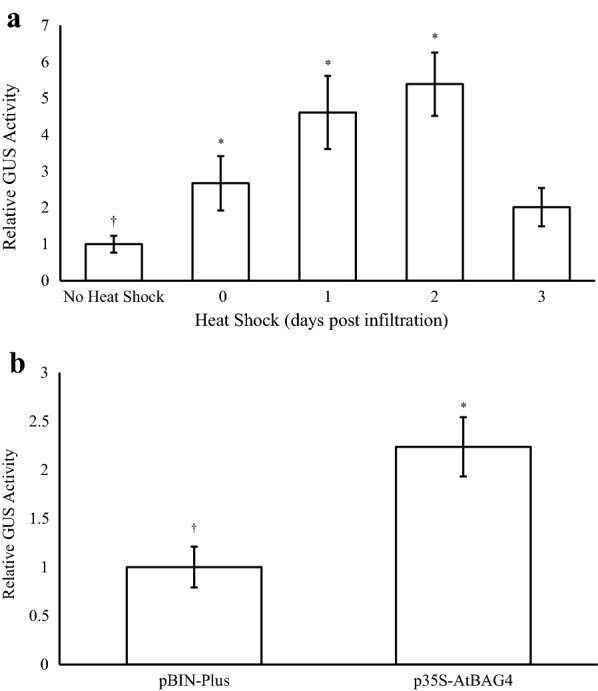
Effects of heat shock treatment and co‐expressing a stress tolerance gene on transient GUS expression. **a** Agrobacteria strain AGL1 harbouring pEAQ‐GSN were infiltrated into *N. benthamiana* leaves and the whole plants heat shocked (37 °C for 30 min), at either 0, 1, 2, or 3 dpi. **b** Co‐transformation with a vector capable of expressing the *Arabidopsis BAG*4 gene product. Leaves were sampled 4 dpi and TSP extracted for GUS fluorometric enzyme assays. Columns represent relative levels of mean GUS enzyme activities and bars represent ± SE. (†) indicates the reference treatment and (*) indicates data significantly different to the reference (*p* < 0.05)

To determine the effects of a physical heat shock on transient expression, whole plants were exposed to a 37 °C heat treatment for 30 min at various time points following agroinfiltration with AGL1 harbouring pEAQ-GSN (Fig. 4a). No significant increases in GUS levels were observed in plants subjected to a heat shock immediately following infiltration (Day 0) or 3 dpi. In contrast, plants heat shocked at either 1 or 2 dpi had significantly higher transient expression, with GUS activities four to fivefold higher than plants that were not heat treated.

### Effects of co-expressing a gene known to confer stress tolerance

*A. tumefaciens* strain AGL1 harbouring the vector pEAQ-GSN were co-infiltrated with agrobacteria containing p35S-AtBAG4 or an empty vector control (pBIN-Plus) and GUS expression measured 4 dpi. Co-expression of the *AtBAG*4 stress tolerance gene significantly increased GUS levels twofold over the empty vector control (Fig. 4b).

### Effects of co-expressing different virus-derived suppressors of PTGS

**Fig. 5:**
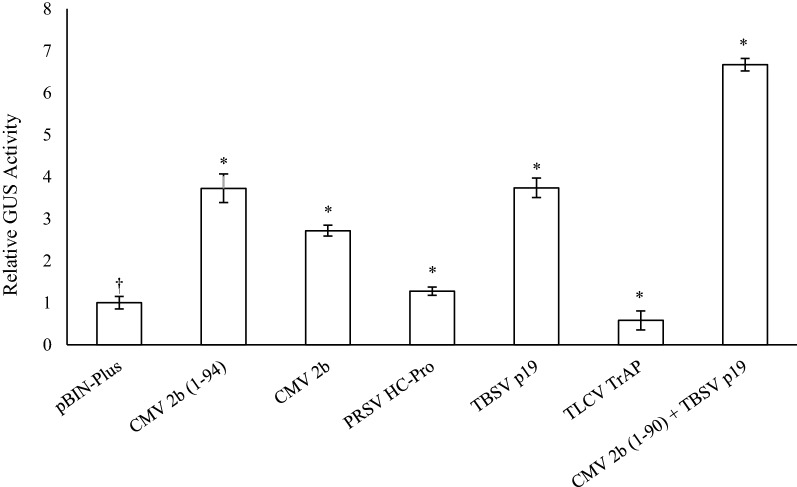
Effects of co‐expressing suppressors of gene silencing on transient GUS expression. Agrobacteria strain AGL1 harbouring the vector p35S‐GSN were co‐infiltrated into *N. benthamiana* leaves with various suppressors of gene silencing, including CMV 2b, CMV 2b (1-94) truncation, PRSV HC-Pro, TBSV p19, TLCV TrAP and both the CMV 2b (1-94) truncation and TBSV p19 together. Leaves were sampled 4 dpi and TSP extracted for GUS fluorometric enzyme assays. Columns represent relative levels of mean GUS enzyme activities and bars represent ± SE. (†) indicates the reference treatment and (*) indicates data significantly different to the reference (*p* < 0.05)

As the pEAQ-HT vector contains an expression cassette encoding the p19 PTGS suppressor, the vector p35S-GSN was used in its place. The effect of co-expressing different suppressors of PTGS on transient GUS expression was examined by co-infiltrating leaves with agrobacteria strain AGL1 harbouring p35S-GSN in combination with one of the following vectors containing a virus-derived suppressor of gene silencing: p35S-TSBV.P19, p35S-CMV.2b, a C-terminal truncated CMV 2b (1-94), p35S-PRSV.HC-Pro, p35S-TLCV.TrAP or an empty vector control (Fig. 5). At 4 dpi, the TBSV p19, CMV 2b, truncated CMV 2b (1-94) and PRSV HC-Pro all significantly increased GUS levels over the empty vector control. Co-expression of either TBSV p19, CMV 2b or the truncated CMV 2b (1-94) resulted in a 2.5 to fourfold increase in GUS levels, while the PRSV HC-Pro had no major effect. Co-expression of TLCV TrAP had an inhibitory effect reducing GUS levels twofold. Co-delivery of both the TBSV p19 and the CMV 2b (1-94) proteins significantly increased GUS activity approximately sixfold.

### Effects of co-expressing virus-derived cell cycle proteins

**Fig. 6:**
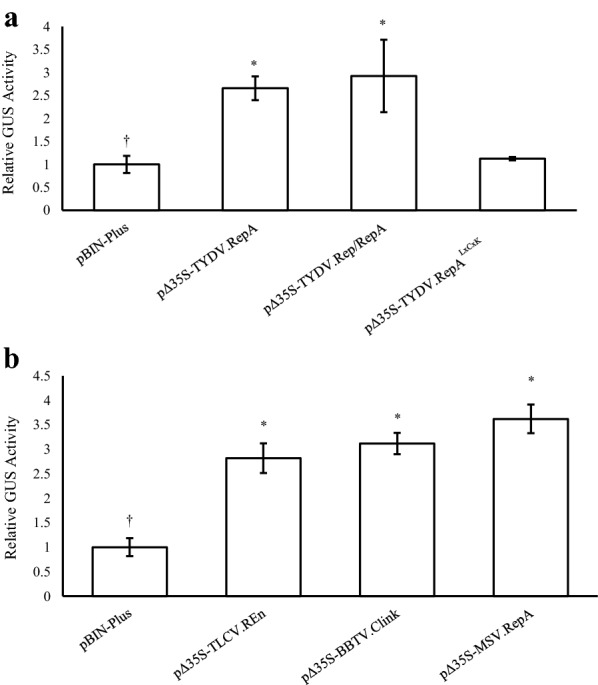
Effects of co‐expressing virus‐derived genes encoding cell cycle regulatory proteins. Agrobacteria strain AGL1 harbouring pEAQ‐GSN were co‐infiltrated into *N. benthamiana* leaves with **a** TYDV *Rep*/*RepA* or *RepA* genes and the TYDV *RepA* gene containing a LxCxK mutation in the RB-binding motif, and **b** cell cycle regulatory genes from related circular ssDNA plant viruses, under the transcriptional control of the truncated CaMV 35S (− 90) promoter (Δ35S). Leaves were sampled 4 dpi and TSP extracted for GUS fluorometric enzyme assays. Columns represent relative levels of mean GUS enzyme activities and bars represent ± SE. (†) indicates the reference treatment and (*) indicates data significantly different to the reference (*p* < 0.05)

Agrobacteria harbouring pEAQ-GSN were co-infiltrated with the TYDV Rep/RepA or TYDV RepA under the transcriptional control of the weaker truncated CaMV (− 90) promoter. At 4 dpi, co-expression of either protein significantly increased base GUS levels about two to threefold (Fig. 6a). To determine whether the LxCxE retinoblastoma-binding motif played a role in this enhancer activity, an E to K mutation was made in the RepA LxCxE motif. Co-expression of the RepA^LxCxK^ mutant failed to elevate GUS levels which were equivalent to those levels afforded by pEAQ-GSN co-infiltrated with the empty vector control pBIN-Plus (Fig. 6a). To investigate whether other cell cycle proteins derived from related circular ssDNA plant viruses could also enhance transient expression levels, the Maize streak virus *RepA*, Tomato yellow leaf curl *REn* and the Banana bunchy top virus *Clink* genes were each placed under the transcriptional control of the CaMV (− 90) promoter. Agrobacteria strain AGL1 harbouring each vector were co-infiltrated with pEAQ-GSN, and GUS activities measured at 0 and 4 dpi (Fig. 6b). Independent co-delivery of all cell cycle proteins resulted in significantly increased base GUS levels of between two and threefold.

### Combining optimal expression elements: pSPECIAL and pNEEDS

**Fig. 7:**
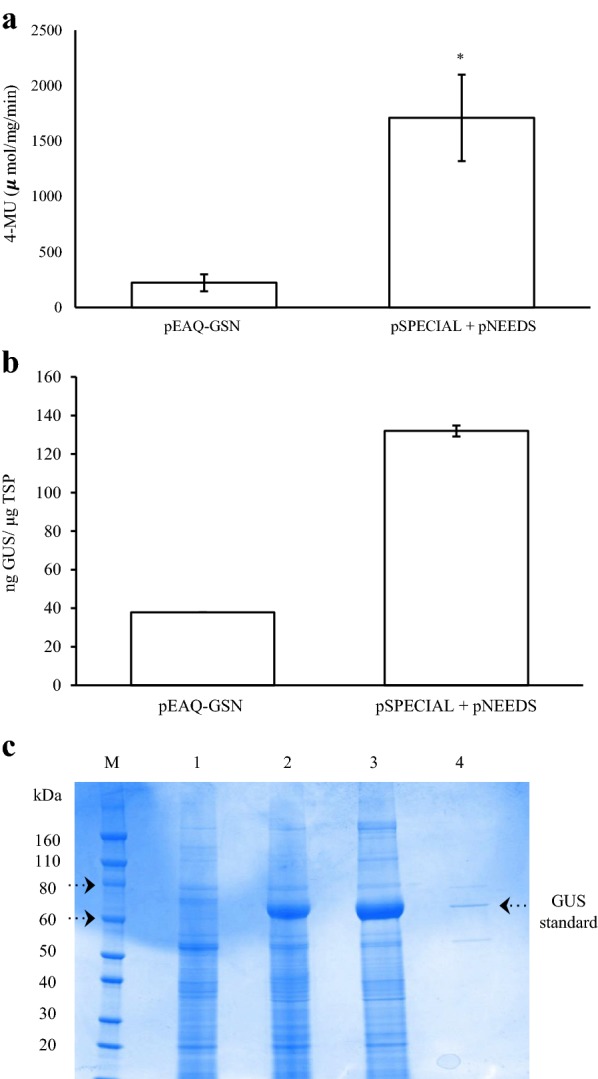
Comparison of GUS expression afforded by pEAQ-GSN versus pSPECIAL + pNEEDS vectors. Agrobacteria strain AGL1 harbouring pEAQ‐GSN or pSPECIAL and pNEEDS were infiltrated into *N. benthamiana.* Leaves were sampled 4 dpi and GUS levels measured by **a** fluorometric MUG enzyme assays, **b** ELISA and **c** SDS‐PAGE. M = Novex^®^ Sharp Pre‐stained Protein Standard; 1 = pBIN‐Plus in MMA; 2 = pEAQ‐GSN in MMA; 3 = pSPECIAL + pNEEDS in MMA‐LP with heat shock (2 dpi); 4 = Purified GUS standard (0.3 μg) (GUS Type VII‐A; Sigma‐Aldrich G7646). Columns in **a** represent relative levels of mean GUS enzyme activitiy (μmol 4-MU/mg protein/min) and bars represent ± SE. (*) indicates a significant difference (*p* < 0.05). Columns in **b** represent mean GUS concentrations and bars represent ± SE

In order to combine the optimal features identified in this study for maximal transient expression, two vectors were assembled (1) pSPECIAL: essentially pEAQ-GSN with the CMV 2b gene under the transcriptional control of the CaMV 35S promoter, and (2) pNEEDS: which contained the *Arabidopsis BAG*4 gene and the TYDV *Rep/RepA* genes under the transcriptional control of the nos and CaMV (− 90) promoters, respectively. For comparison, *N. benthamiana* plants were infiltrated with agrobacteria (strain AGL1) harbouring pEAQ-GSN in standard MMA as described by Sainsbury et al. [31]. Vectors pSPECIAL and pNEEDS were co-infiltrated (1:1 ratio) in an optimised infiltration media MMA-LP (MMA containing 500 μM acetosyringone, 5 μM α-Lipoic acid, and 0.002% Pluronic F-68). In the latter case, plants were heat shocked at 37 °C at two dpi. At four dpi, GUS activity was measured using fluorometric assays (Fig. 7a). GUS levels afforded by the vectors pSPECIAL and pNEEDS were significantly higher (approximately 6 to 8-fold) than that directed by pEAQ-GSN.

ELISA was also used to quantify the absolute amount of recombinant GUS produced by both expression platforms. At 4 dpi, extracts from leaves infiltrated with pEAQ-GSN yielded, on average, approximately 37.8 ng GUS/μg TSP compared to 132 ng GUS/μg TSP from the pSPECIAL and pNEEDS vectors (Fig. 7b). Based on these ELISA readings, the amount of recombinant GUS generated was significantly greater (about 3.5-fold) than the pEAQ-HT system and represented approximately 13% of leaf TSP.

TSP extracts were electrophoresed through acrylamide and stained with Coomassie Brilliant Blue dye (Fig. 7c). A dense band of approximately 70 kDa, was present in TSP extracts from both pEAQ-GSN and pSPECIAL and pNEEDS vectors, but not in TSP isolated from the leaves infiltrated with the empty vector. Based on size (GUS is approximately 68.28 kDa in mass) and the co-migration of the bands with the GUS standard, these bands were assumed to represent plant-made recombinant GUS enzyme.

## Discussion

Agroinfiltration has become the method of choice to rapidly express recombinant biologics such as therapeutics and vaccine candidates and to study gene function, gene silencing or gene-for-gene interactions *in planta*. To maximize expression levels, researchers have sought to optimize the process at many levels, for example by increasing bacterial transformation rates, tailoring the vector to contain virus-derived elements that increase transgene transcription/translation and minimize PTGS, and by utilizing host species that are highly amenable to the infection process and support high levels of recombinant protein accumulation. While these advances have assisted in developing agroinfiltration as a cost-effective protein production platform, basic aspects of the process have not been fully explored. In this study, agroinfiltration of *N. benthamiana* with the pEAQ-HT vector system [31, 32] was used to define and optimize key elements necessary for rapid, high-level, transient gene expression.

Genetic background of the *Agrobacterium* can greatly influence the ability of the phytopathogen to act as a vehicle for T-DNA transfer. As such, three common laboratory strains were tested for their ability to support transient GUS expression in *N. benthamiana*. These strains represented three of the four opine utilising types; octopine (LBA4404), nopaline (C58C1) and succinamopine (AGL1) and members originated from either of the wildtype progenitor isolates C58 (AGL1 and C58C1) and Ach5 (LBA4404). Bacterial density during the infection process can also affect transformation efficiencies; too dilute a culture may result in a low bacteria/target cell ratio thereby decreasing transformation frequencies, whereas concentrated bacterial cultures can lead to bacterial overgrowth and cause excessive tissue damage [5, 40, 41]. Of the *Agrobacterium* strains tested, the hypervirulent strain AGL1 yielded the highest GUS activity 4 dpi, suggesting AGL1 has a more aggressive disposition for infection or perhaps a more effective bacteria-encoded T-DNA transfer mechanism in comparison to the other two strains [42, 43]. AGL1 cell culture densities between OD_600_ = 0.1 and 1.5 provided the highest transient GUS activities and were not significantly different. Accordingly, agrobacteria strain AGL1 at a density of OD_600_ = 1.0 were used routinely for all further experimental comparisons.

It is well understood that the wound-induced, plant phenolic signal chemical acetosyringone plays an important role in both chemotaxis and the induction of *Agrobacterium* virulence (*vir*) genes [44]. Incorporation of acetosyringone in the co-cultivation media during bacterial infection has reportedly enhanced the transformation rates of many plant species including those previously considered recalcitrant to transformation [45] and, in some cases, broadened the host range of the *Agrobacterium* strain itself [46]. Agroinfiltration is a relatively non-invasive procedure with cell damage often limited to the site of injection. As such, conditioning of the bacteria with acetosyringone prior to delivery is likely important in the absence of wounding. Increasing acetosyringone concentrations in the infiltration media resulted in a proportional increase in reporter gene expression, peaking at a concentration of 500 μM. A similar correlation between acetosyringone levels and transient expression was observed by Wydro et al. [12]. Other phenolic compounds, such as vanillin and cinnamic acid, have also been shown to strongly induce *vir* genes [47] and, therefore, may warrant further investigation as potent chemical alternatives.

Plant defence mechanisms in response to pathogen invasion often generate an oxidative burst and the induction of pathogenesis-related genes, resulting in necrosis and cell death at the point of infection. In order to prevent this during *Agrobacterium*/plant interaction, many chemical additives have been tested for their ability to suppress oxidative stress, minimise necrosis and increase transformation efficiency. Such additives have included PVP, dithiothreitol, glutathione, ascorbic acid, cysteine, sodium thiosulfate, sodium selenite and DL-α-tocopherol [16, 19, 48]. In this study, the effects of incorporating three different antioxidant compounds, lipoic acid, ascorbic acid, and PVP, in the infiltration media used to deliver agrobacteria were compared. Of these, lipoic acid had the greatest effect with a concentration of 5 µM resulting in a sixfold increase in transient GUS activity compared to the control. Lipoic acid is a sulphur-containing compound that exists in nature as a metabolic antioxidant capable of scavenging reactive oxygen species and recycling other antioxidants [49]. The compound has successfully been used to increase the frequency of *Agrobacterium*-mediated transformation of a number of crops including soybean, tomato, wheat and cotton [19]. In tomato, the compound was shown to markedly reduce browning in plant tissues following infection and increase the percentage of explants displaying transient reporter expression by threefold. Ascorbic acid has been shown to minimise the secretion of wound-induced phenolics and prevent oxidative stress in rice and peanut transformation [20, 50, 51]. In this work, the chemical did not significantly increase *Agrobacterium*-mediated transient GUS activity, however, its addition has been beneficial in other transient studies [52]. Considering this, we assume that the effects of these chemicals are dependent on bacteria and host plant compatibility factors and their efficacy will most likely vary between studies.

The addition of surfactants such as Silwet-L77, Tween 20 and Pluronic F-68 during co-cultivation has been shown to increase *Agrobacterium*-mediated transformation efficiencies in various crops including wheat [53, 54], *Arabidopsis* [55], banana [23], radish [56] soybean [48] and switchgrass [57]. While it is unclear exactly how these compounds function, it is presumed they reduce the surface tension of the co-cultivation media and perhaps eliminate certain substances that inhibit cell attachment to improve bacterial invasion and ultimately T-DNA delivery [43, 53, 58]. Similar to the studies above, very low concentrations (0.002%) of the surfactant Pluronic F-68 used here were found to improve agroinfiltration, increasing GUS activity twofold.

A physical heat shock to the entire *N. benthamiana* plant 1–2 days following agroinfiltration generated a significant (four to fivefold) increase in transient GUS activity. It is well known that heat shock proteins and chaperones are up-regulated in response to extreme heats and other abiotic stresses to maintain cellular homeostasis [59]. Such proteins facilitate the correct conformational folding of native proteins by binding to the reactive surfaces of partially folded proteins and effectively sequestering their active sites. This limits interactions between partially folded intermediates, prevents aggregation and the degradation of terminally misfolded proteins effectively protecting them from oxidative stress [60–64]. While the heat shock of seedlings and embryogenic cells has been shown to increase *Agrobacterium*-mediated transformation frequencies in crops such as switchgrass [57], banana [24] rice and maize [65], we believe this is the first report of such a treatment for the improvement of agroinfiltration-based transformation/expression in mature plants.

*BAG* genes are an evolutionarily conserved family of multifunction co-chaperone proteins with roles in the promotion of cell survival [66]. *AtBAG*4 is one of seven *BAG* family homologues identified in *Arabidopsis thaliana.* Transgenic *Arabidopsis BAG*4 knockouts display early senescence and unique phenotypes suggesting the gene product is important for normal plant growth and development. Further, over-expression of *AtBAG*4 in tobacco, tomato and banana has been shown to increase tolerance to various biotic and abiotic stresses [67]. In the current study, co-expression of *AtBAG*4 increased GUS levels twofold suggesting this protein may function to reduce the programmed cell death response associated with incompatible *Agrobacterium*/host interaction.

It is well accepted that low transient heterologous gene expression is often the result of PTGS and this bottleneck can be overcome by the co-expression of PTGS suppressors [68]. Many plant viruses encode gene products that are capable of suppressing PTGS, however, their mode of action and potency can vary between virus families. To determine the most effective virus-derived silencing suppressor for our purposes, genes were compared from members of four different virus families, including the *Bromoviridae* (CMV 2b), *Potyviridae* (PRSV HC-Pro), *Tombusviridae* (TBSV p19), and the *Geminiviridae* (TLCV TrAP). Of these, the truncated CMV 2b and TBSV p19 gene products were the most effective at suppressing PTGS, both significantly increasing GUS levels approximately fourfold. TBSV p19 has long been considered a potent suppressor of gene silencing and has been shown to increase transient expression levels in various plant species by sequestering siRNA and preventing their association with the RISC complex [69–72]. In contrast, CMV 2b is able to directly interact with both the RNA and protein components of the silencing pathway [73, 74]. Co-infiltration of both truncated CMV 2b with TBSV p19 effectively doubled GUS activity levels compared to using either silencing suppressor alone. This may indicate that by combining the diverse functions of both proteins i.e. binding and sequestering of siRNAs, preventing siRNA duplex assembly into the RISC, and direct interference with the AGO containing RISC, serves to collectively enhance PTGS suppression.

Geminivirus replication is strongly dependent on the host cell’s DNA synthesis machinery. As such, these viruses, and the related nanoviruses, have developed a means of overcoming cellular quiescence by subverting the cell cycle control mechanism and synchronizing cells to S‐phase, a phase in which host cell DNA polymerases are most abundant. Virus-encoded gene products are thought to achieve this by specifically binding retinoblastoma‐related protein (RBR), a key regulator of the cell cycle, and disrupting the RBR‐E2F complex thereby causing premature entry into S-phase. In mastreviruses and nanoviruses, this interaction occurs through a conserved canonical LxCxE motif in the RepA and Cell cycle link (Clink) proteins, respectively. In other geminiviruses, Rep and REn bind RBR and other cell cycle proteins, such as PCNA, via a different motif [75]. Expression of the Wheat dwarf mastrevirus RepA protein has been shown to increase transformation frequencies in maize callus, suggesting an S-phase transition is also beneficial for *Agrobacterium*-mediated transformation. However, we and others have observed that over-expression of some geminivirus Rep and RepA genes can be phytotoxic and there are few reports of transgenic plants constitutively expressing these gene products [76–79]. As such, the virus genes tested in this study were placed under the transcriptional control of the truncated CaMV 35S (− 90) promoter which has approximately fivefold lower relative activity to that of the complete 35S RNA promoter [80]. Co-expression of cell cycle reprogramming proteins derived from three different circular ssDNA plant viruses all increased transient GUS expression levels and, in the case of the TYDV RepA protein, this activity was shown to be directly dependent on a functional LxCxE motif.

By combining the most effective features into a single expression platform, we aimed to greatly improve expression levels afforded by the pEAQ-HT vector system [31]. The result was a dual vector co-delivery system that incorporated an optimised infiltration medium and heat shock treatment to the whole plant following agroinfiltration. GUS levels afforded by this platform were between six and eightfold higher as estimated by GUS enzyme activity and 3.5-fold higher as estimated by ELISA quantification of absolute GUS protein levels. While the system generated very high GUS levels, this increase does not reflect the sum benefits of individual molecular features or treatments when tested independently. It is possible that some elements when provided in combination may negatively impact transgene expression, perhaps for example the TYDV Rep/RepA phytotoxic gene products. Alternatively, hyperexpression of each gene may simply deplete the host cellular transcription and translation machinery, thus compromising GUS levels. With further refinement, we anticipate expression levels afforded by this system could be further increased. In addition, valuable aspects of this study alone could be incorporated into other expression platforms as a simple means of enhancing protein production capacities in plants.

## Additional files

**Additional file 1.** List of primer used in this study.

**Additional file 2.** Description of vectors used in this study.

## Abbreviations

*BAG*: Bcl-2 associated athanogene
*CaMV*: cauliflower mosaic virus
*CMV*: cucumber mosaic virus
*CPMV*: cowpea mosaic virus
*dpi*: days post infiltration
*EAQ*-*HT*: easy and quick-hyper translatable
*GUS*: β-glucuronidase
*Hsp*: heat shock protein
*MMA*: MES; MgCl_2_; acetosyringone (infiltration buffer)
*MSV*: maize streak virus
*MU*: 4-methylumbelliferone
*MUG*: 4-methylumbelliferyl-β-D-glucuronide trihydrate
*nos*: gene encoding nopaline synthase
*PVP*: polyvinylpyrrolidone
*PRSV*: papaya ringspot virus
*PTGS*: post transcriptional gene silencing
*REn*: replication enhancer protein
*Rep*: replication associated protein
*RepA*: replication associated protein A
*SE*: standard error
*TBSV*: tomato bushy stunt virus
*TYDV*: tobacco yellow dwarf virus
*TLCV*: tomato leaf curl virus
*TrAP*: transcriptional activator protein
*TSP*: total soluble protein
*uidA*: reporter gene encoding β-glucuronidase
