# Supplementary material for: Improving agroinfiltration-based transient gene expression in Nicotiana benthamiana
Source: Plant Methods. 2018 Aug 25;14:71. doi: 10.1186/s13007-018-0343-2 (PMC6109318; doi:10.1186/s13007-018-0343-2)
Supplement: Supplementary file 1 — Additional file 1. List of primer used in this study. [file 13007_2018_343_MOESM1_ESM.docx]

| Vector Name | Promoter | Gene  (GenBank Accession) | Terminator | PCR primers used in assembly | Gene template/ source | Restriction sites used in assembly |
| --- | --- | --- | --- | --- | --- | --- |
| pEAQ-HT | CaMV 35S (+CPMV 5’ UTR) | - | nos (+CPMV 3’ UTR) | - | Ref [32] | - |
| pEAQ-GUS | CaMV 35S (+CPMV 5’ UTR) | *uid*A | nos (+CPMV 3’ UTR) | - | p35S-GSN  Ref [33] | AgeI/XhoI  BamHI(blunt)/SalI |
| p35S-AtBAG4 | CaMV 35S | At BAG4  (NM_115037.7) | nos | At_BAG4-F and At_BAG4-R | *Arabidopsis thaliana* cv. Landsberg | AsiSI/SacI |
| p35S-TBSV.p19 | CaMV 35S | TBSV p19 (M21958.1) | nos | TBSVp19-F and TBSVp19-R | Tomato bushy stunt virus | AsiSI/SacI |
| p35S-CMV.2b | CaMV 35S | CMV 2b (AB506799.1) | nos | CMV2b-F and CMV2b-R | Cucumber mosaic virus | AsiSI/SacI |
| p35S-PRSV.HC-Pro | CaMV 35S | PRSV HC-Pro (JQ394692.1) | nos | PRSVHCPro-F and PRSVHCPro-R | Papaya ringspot virus | AsiSI/SacI |
| p35S-TCLV.TrAP | CaMV 35S | TLCV *TrAP* (NC_003896.1) | nos | TLCVTrAP-F and TLCVTrAP-R | Tomato leaf curl virus | AsiSI/SacI |
| p35S-CMV.2b (1-94) | CaMV 35S | CMV 2b (AB506799.1) | nos | CMV2b-F and CMV2b-R(1-94) | Cucumber mosaic virus | AsiSI/SacI |
| p35S-TYDV.Rep/RepA | CaMV 35S | TYDV (M81103.1) | nos | TYDVRep-Ex1-F and TYDVRepA-R | pDH51  Ref [33] | EcoRI/XbaI |
| p35S-TYDV.RepA | CaMV 35S | TYDV (M81103.1) | nos | TYDVRepA-F and TYDVRepA-R | pDH51  Ref [33] | EcoRI/XbaI |
| p35S-TLCV.REn | CaMV 35S | TLCV Ren (NC003896.1) | nos | 35S‐F and TCLVREn‐R | p35SAUSREN [35] | AscI/XbaI |
| p35S-BBTV.Clink | CaMV 35S | BBTV *Clink* (L41578.1) | nos | 35S‐F and BBTVClink‐R | p35S‐BBTV.ORF5 | AscI/XbaI |
| p35S-MSV.RepA | CaMV 35S | MSV (AY138520) | nos | - | chemically synthesised by GeneArt | AscI/SacI |
| pΔ35S-TYDV.Rep/RepA | CaMV Δ35S | - | nos | - | p35S-TYDV.Rep/RepA | SmaI/PacI  EcoRV/PacI |
| pΔ35S-TYDV.RepA | CaMV Δ35S | - | nos |  | p35S-TYDV.RepA | SmaI/PacI  EcoRV/PacI |
| pΔ35S-BBTV.Clink | CaMV Δ35S | - | nos |  | p35S-BBTV.Clink | SmaI/PacI  EcoRV/PacI |
| pΔ35S-TLCV.REn | CaMV Δ35S | - | nos |  | p35S-TLCV.REn | SmaI/PacI  EcoRV/PacI |
| pΔ35S-MSV.RepA | CaMV Δ35S | - | nos |  | p35S-MSV.RepA | SmaI/PacI  EcoRV/PacI |
| pΔ35S-TYDV.RepA^LxCxK^ | CaMV Δ35S | - | nos | Δ35S-F; TYDV^LxCxK^mut-R, TYDV^LxCxK^mut-F and TYDVRepA-R2 | pΔ35S-TYDV.RepA | AscI/SacI |
| pSPECIAL | CaMV 35S (+CPMV 5’ UTR)  CaMV 35S  CaMV 35S | GUS  TBSV p19  CMV 2b (1-94) | nos (+CPMV 3’ UTR)  CaMV 35S  nos | 35S_FseI-F and nosT-FseI-R | pEAQ-GUS  p35S-TBSV.p19  p35S-CMV.2b (1-94) | FseI |
| pNEEDS | nos  CaMV Δ35S | At BAG4  TYDV Rep/RepA | nos  nos | nosP_NheI-F and nosP_AsiSI-R | p35S-AtBAG4  pΔ35S-TYDV.Rep/RepA | EcoRI  NheI/AsiSI |
