# Supplementary material for: Improving agroinfiltration-based transient gene expression in Nicotiana benthamiana
Source: Plant Methods. 2018 Aug 25;14:71. doi: 10.1186/s13007-018-0343-2 (PMC6109318; doi:10.1186/s13007-018-0343-2)
Supplement: Supplementary file 2 — Additional file 2. Description of vectors used in this study. [file 13007_2018_343_MOESM2_ESM.docx]

Additional Data File 2: List of primers used in this study

| Target Sequence | Primers | Sequence (5’🡪3’) |
| --- | --- | --- |
| *AtBAG*4 | At_BAG4-F | CGGCGATCGCATGATGCATAATTCAACCGAAG |
|  | At_BAG4-R | GCGAGCTCTCAGTCAAATTTCTCCCAATCTTG |
| TBSV *p19* | TBSVp19-F | GCGCGATCGCCATGGAAAGGGCTATTCAGGGAAATGATGC |
|  | TBSVp19-R | CGGAGCTCCCCTTACTCGCTTTCTTTTTCG |
| CMV *2b* | CMV2b-F | GCGCGATCGCATGGAATTGAACGTAGGTGC |
|  | CMV2b-R | GCGAGCTCTCAGAAAGCACCTTCCGCCCATTCG |
| CMV *2b* (1-94) | CMV2b-F | GCGCGATCGCATGGAATTGAACGTAGGTGC |
|  | CMV2b-R (1-94) | CGGAGCTCGTCAGAATCTTAGTCTTCCGCCGAT |
| PRSV *HC-Pro* | PRSVHCPro-F | CGGCGATCGCGCAGTACAATGACGTGGCTGAAAAATTCTGGC |
|  | PRSVHCPro-R | CGGAGCTCCCGATGTAGTGCTTCATTTCACTATCG |
| TLCV *TrAP* | TLCVTrAP-F | GCGCGATCGCATGCAGAATTCATCACCCTC |
|  | TLCVTraP-R | GCGAGCTCTTAAATACCCTCAAGAAACGCC |
| TYDV Rep/RepA | TYDVRep-Ex1-F | TCTAGAGTCGAGTTAATTGCTTCCAGAGTGGGACGAATTTGAAG |
| TYDV *RepA* | TYDVRepA-F | GAATTCATGCCTTCAGCCCCCCAGAAAACCAAATCCTTC |
|  | TYDVRepA-R | TCTAGAGTCGAGTTAATTGCTTCCAGAGTGGGACGAATTTGAAG |
| CaMV 35S promoter + BBTV *Clink* | 35S-F | GGCGCGCCCATGGAGTCAAAGATTCAAATAGAGGA |
|  | BBTVClink-R | TCTAGAGTCGAGTTAGAGTAATGTTACATCATAGTCTGATATAAC |
| CaMV 35S promoter + TLCV *REn* | 35S-F | GGCGCGCCCATGGAGTCAAAGATTCAAATAGAGGA |
|  | TLCVREn-R | TCTAGAGTCGAGTTAATAAAAATTAAATTTTATATCATGATC |
| TYDV *RepA* LxCxK  mutant - overlapping PCR | Δ35S-F | GGCGCGCCgatatctccactgacgtaagggatgacg |
|  | TYDV^LxCxK^mut-R | GGTCTCTCCATAACTGCAGATCTTTATGACATTGGAG |
|  | TYDV^LxCxK^mut-F | CATTCACTGAGGAAGATCTCCAATGTCATAAAGATCTGC |
|  | TYDVRepA-R2 | gagctcttaattgcttccagagtgggacgaa |
| CaMV 35S promoter-  CMV *2b*-nosT cassette | 35S_FseI-F | GCGGCCGGCCTCATGGAGTCAAAGATTCAAATAGAGGACC |
|  | nosT_FseI-R | CGGGCCGGCCCCCGATCTAGTAACATAGATGACACCGCGCGCG |
| nos promoter | nosP_NheI-F | CCGCGATCGCAGATCCGGTGCAGATTATTTGGATT |
|  | nosP_AsiSI-R | CAGCTAGCAAATATTTCTTGTCAAAAATGCTCCA |
